# Target Body Temperature in Very Low Birth Weight Infants: Clinical Consensus in Place of Scientific Evidence

**DOI:** 10.3389/fped.2019.00227

**Published:** 2019-06-07

**Authors:** Anna Perez, Frauke van der Meer, Dominique Singer

**Affiliations:** Division of Neonatology and Pediatric Critical Care Medicine, Center of Obstetrics and Pediatrics, University Medical Center Hamburg-Eppendorf, Hamburg, Germany

**Keywords:** preterm neonates, VLBWI, thermal care, NICU, body temperature

## Abstract

**Background:** Although thermal care is part of the daily routine in Neonatal Intensive Care Units (NICUs), scientific evidence on what is the appropriate body temperature for very low birth weight infants (VLBWI) is largely lacking.

**Aim:** To find out to what extent the standards of thermal care vary among high-level NICUs, especially with respect to the target body temperature in VLBWI.

**Methods:** An online survey with 21 questions on thermal care in three categories of VLBWI was sent to 149 NICUs in Germany, Switzerland, and Austria.

**Results and discussion:** Out of 112 (75%) returned questionnaires, 87 (58%) were included into analysis. A significant increase in incubator settings (air temperature/relative humidity) with decreasing gestational age and birth weight was reported, according to common textbook recommendations. However, a uniform target body temperature of 36.99 ± 0.19°C was chosen for all VLBWI categories. Likewise, the cut-off points for hypo- and hyperthermia were defined very similarly and showed low inter-center variability. This is a remarkable finding in view of the fact that the body temperature of mammalian fetuses *in utero* is 0.5–1.0°C higher than that of the mother.

**Conclusion:** Despite lacking scientific evidence, there is a tacit consensus among high-level NICUs that 37.0°C is the appropriate body temperature in VLBWI, regardless of gestational age and birth weight. As this is below the intrauterine “breeding temperature” of the fetus, further research on this topic is warranted.

## Introduction

Body temperature control is of outmost importance in preterm neonates. Both hypo- and hyperthermia are known to negatively affect their short- and long-term outcome. Therefore, considerable efforts are made in neonatal care to keep the babies' body temperature in the “normal” range. However, scientific evidence on what might be the “normal” body temperature in preterm neonates is largely lacking.

*In utero*, the fetal temperature is higher than the maternal temperature (1–3). For term babies the World Health Organization (WHO) recommends a body temperature range of 36.5–37.5°C (4). Some authors suggest to apply the same reference range to preterm infants, too (5, 6). However, most clinical studies on preterm infants' heat balance have been published decades ago when limitations in neonatology were much greater than they are today (7–10). Moreover, the majority of papers on neonatal thermoregulation deal with body temperature on admission or during the first hours of life and its influence on short-term outcome parameters such as mortality (5, 6, 11, 12). Very little, if any, is known about the optimal target body temperature of preterm infants during their stay on the Neonatal Intensive Care Unit (NICU), and its potential influence on long-term outcome parameters such as neurological development. Practical recommendations on how to adjust the ambient conditions for preterm neonates, in order to provide “thermoneutral care,” are given in relevant textbooks (13–15). Nevertheless, in a French survey published in 2012 including a total of 186 NICUs it was shown that the variability of thermal management in daily routine was still large and often not adapted to the infants' age and maturity. Primary incubator settings for temperature and relative humidity were distributed very heterogeneously (16).

In view of these findings, we conducted a survey to assess routine practices of thermal care and, above all, body temperature targets in very low birth weight infants (VLBWI) among high-level NICUs in Germany, Switzerland, and Austria.

## Materials and Methods

The survey instrument was a specifically designed online questionnaire. The software tool Survey Monkey^®^ was used for survey design, distribution, and recollection of data.

**Figure 1:**
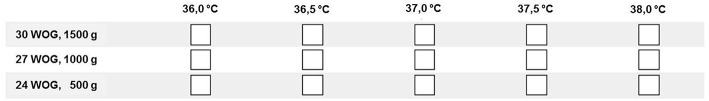
Sample question from the online questionnaire on thermal management in three different categories of VLBWI (Question #6: Target body temperature): Participants were asked to check all boxes/temperatures that would be deemed acceptable on their NICU.

The questionnaire consisted of 21 questions covering the following items: demographics, temperature target range, temperature measurement methods, heat therapy devices including mode of operation, standards and complementary methods of thermal care. Within the demographics section, the size of the NICU and the number of infants < 1,500 g admitted per year were recorded as well as the staff position of both the answering participant and the caretaker primarily responsible for thermal management on the specific NICU. With regard to the temperature target range, body core temperature and definitions of hypo- and hyperthermia were addressed. Here, temperature range and cut-off points for hypo- and hyperthermia were provided in predefined temperature intervals of 0.5°C. For further specification in the temperature target range and heat therapy devices sections of the survey, the neonates were categorized into three exemplary subgroups according to their gestational age (GA) and birth weight (BW): (1) 30 weeks of gestation (WOG)/1,500 g, (2) 27 WOG/1,000 g, (3) 24 WOG/500 g (Figure 1; cf. also Data Sheets 1 and 2 in the Supplementary Material).

A pretest of the survey in order to evaluate its comprehensibility and feasibility was run among the medical and nursing staff of the Division of Neonatology and Pediatric Critical Care Medicine in our institution. The finalized survey version was sent out between October 2014 and January 2015 and was addressed to the respective head neonatologists of 149 perinatal tertiary care centers, including 136 German, six Austrian, and seven Swiss centers. It was her/his decision to answer the survey by herself/himself or to give it to one of the department's staff physicians, head nurses or certified nurses. Only one member of each center was allowed to answer the survey, regardless of its professional group (head neonatologist, staff physician, head nurse, certified nurse). Selection of participants was conducted via an internet-based systematic research. In Germany, perinatal “level one” centers are defined as centers which regularly care for infants <1,250 g and which provide a minimum of six NICU beds including respiratory support options. Only perinatal centers “level one” were included as participants in Germany. To ensure comparability all Austrian and Swiss centers were selected by applying the same criteria. A maximum of two electronic reminders were sent every 15 days to all centers with a pending answer. Only one answered survey per participating center was eligible for analysis. Eligibility for analysis was given if more than the first five questions of the survey were answered.

### Statistical Analysis

We used chi-squared tests to compare qualitative variables and an analysis of variance to test quantitative variables with the use of a *post-hoc* Fisher's protected least square difference test if F-values were significant. Pearson coefficients (*r*) were calculated for the correlation between selected variables. The statistical significance threshold was set to *p* < 0.05. Data are presented as counts and percentages or as mean ± SD, and inter-level variability is expressed as the coefficient of variation (CV) which is SD divided by the mean. All analyses were performed using SPSS 22 (IBM Corp., Armonk, NY, USA).

## Results

The survey return rate was 75% (*n* = 112). A total of 25 answered questionnaires (22%) had to be excluded from analysis. Reasons for exclusion were: multiple answers from the same participating center (*n* = 21) and incomplete response (less than first five survey questions answered) (*n* = 3). Since only two out of six Austrian NICUs returned an answered questionnaire of which one contained an incomplete response, the analysis was confined to German and Swiss centers. Finally, 87 returned questionnaires were included into statistical analysis (58%). Of those, 82 (94%) contained a complete and five (6%) an incomplete response.

The mean size of the participating NICUs amounted to 16.2 ± 6.6 (median 15) beds, the mean number of treated VLBWI to 59.2 ± 41.8 (median 50) neonates per year. The survey was mostly answered by the head neonatologist (*n* = 64; 74%) or the head nurse (*n* = 11; 13%) of the respective department whereas in the majority of centers, the temperature management was performed by the nursing staff at bedside (*n* = 76; 87%) (Table 1). Almost half of the centers (*n* = 34; 41%) based their thermal management decisions on clinical bedside assessment, most others (*n* = 37; 45%) report the use of standard operating procedures (SOPs).

**Table 1:** Demographics of the online survey: The questionnaire was mainly filled by head physicians or head nurses whereas thermal management is mostly performed by (certified) nurses at bedside.

	**Staff position of survey participant *n* (%)**	**Staff member mainly responsible for thermal management *n* (%)**
Head physician	64 (74)	4 (5)
Ward physician	5 (6)	3 (3)
Head nurse	11 (13)	4 (7)
(Certified) nurse	7 (8)	76 (87)

In the majority of centers (*n* = 60; 69%) temperature measurement was conducted continuously, mostly by using a skin probe either attached to the back/lying surface (43%) or to the anterior abdomen of the infant (28%). Rectal probes were less frequently used for continuous temperature monitoring (8%). Gradient measurement, measuring the gradient between the central and peripheral body temperature with a sensor placed on the anterior abdomen and another on the sole of foot, was only used by 16% of the centers. Thirty-one percent of the centers reported the use of intermittent rectal temperature measurement using a digital thermometer.

**Figure 2:**
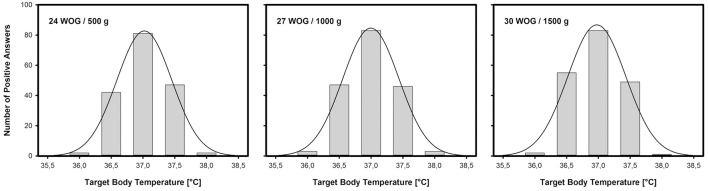
Target body temperatures for preterm neonates on high-level NICUs: Number of positive answers and normal distribution in three different categories of VLBWI. As in term babies, 37.0°C is presumed to be the normal body (core) temperature for VLBWI, nearly irrespective of gestational age (in Weeks Of Gestation, WOG) and/or birth weight (in grams).

The reported target body temperature range was similar across all GA and BW categories and amounted to 37.02 ± 0.2°C (24 WOG/500 g), 36.99 ± 0.22°C (27 WOG/1,000 g), and 36.98 ± 0.17°C (30 WOG/1,500 g), respectively (mean ± SD), with a common average of 36,99 ± 0,19°C (Figure 2). Inter-center variability was low with a variation coefficient of 1%. Overall, if answering, the head nursing staff designate a higher target range for body temperature in preterm infants compared to the answering head physicians (*p* = 0.003) or answering staff physicians (*p* = 0.015).

**Figure 3:**
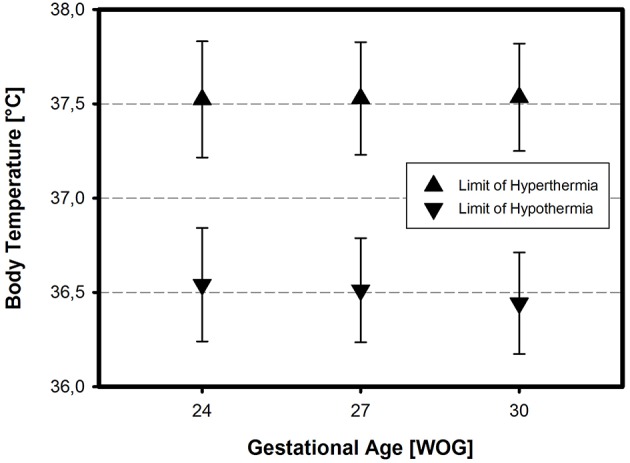
Definition of hypothermia and hyperthermia in three different categories of VLBWI. The respective temperature limits (means ± SD) reflect the assumption that 37.0°C is normal, independent of gestational age and/or birth weight (in Weeks Of Gestation, WOG)—except for a slightly higher limit of hypothermia in the most immature neonates.

Hypothermia was defined homogenously across all three preterm infant categories, with a non-significant trend toward a higher temperature cut-off point in the smallest category of preterm infants (24 WOG, 500 g) compared to infants of 30 WOG, 1,500 g (Figure 3). Again, inter-center variability was low with a variation coefficient of 1%. Answering head nurses (*p* = 0.001) and certified nurses (*p* = 0.04) define a higher temperature value as hypothermia than the answering head physicians (means: 36.65 and 36.60°C, respectively, vs. 36.46°C). If thermoregulatory decision-making is based on clinical bedside assessment only, hypothermia was defined earlier, i.e., at higher temperature values than when based on recommendations from the scientific literature (*p* = 0.007).

Hyperthermia was defined similarly across all centers (variation coefficient 1%), and was equally independent of the infant's GA and body BW (Figure 3; cf. also Table S1 in the Supplementary Material). If the thermoregulatory decision-making was based on clinical bedside assessment only, hyperthermia was considered earlier, i.e., at lower temperature values, compared to the use of SOPs (*p* = 0.002).

All participating centers used the incubator rather than the heat radiator as heat therapy device. Sixty-eight percent (*n* = 59) prefer the air temperature control mode (ATC) to the skin servo-control mode (SSC) (*n* = 27; 31%) in routine practice.

**Figure 4:**
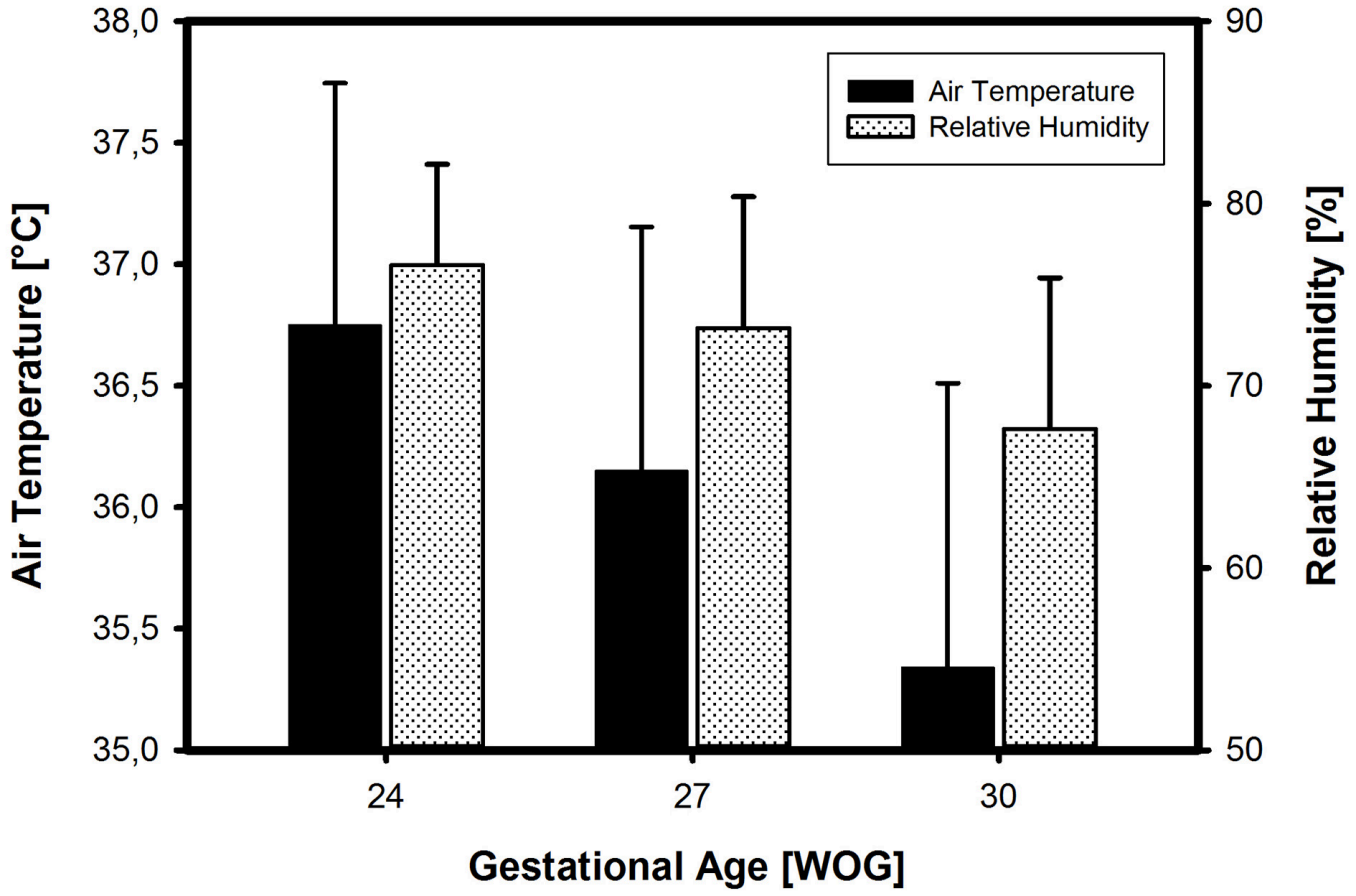
Incubator settings (on admission to the NICU) in three different categories of VLBWI. There is a clear increase in both air temperature and relative humidity (means ± SD) with decreasing gestational age (in Weeks Of Gestation, WOG) and/or birth weight, in accordance with common textbook recommendations.

In the *ATC mode of incubator care*, the air temperature on admission depended on the infant's GA and BW. The smaller and more immature the infants, the higher the selected air temperature levels. The inter-center variability was 3%. The relative humidity on admission also depended on the infant's GA and BW, with higher humidity levels being chosen in smaller and more immature infants. These differences were highly significant (*p* ≤ 0,001) (Figure 4). There was a significant positive correlation (*r* = 0.385, *p* = 0.004) between ambient temperature and relative humidity in the most immature babies (24 WOG/500 g) indicating that those, who prefer higher ambient temperatures, also aim at higher relative humidities. The inter-center variability for incubator humidity levels was high with a variation coefficient of 11%, highest for infants of 30 WOG/1,500 g with a variation coefficient of 12%. Smaller centers (≤50 VLBWI per year) chose significantly lower humidity levels than centers with higher patient volume (*p* = 0.004).

In the *SSC mode of incubator care*, the air temperature on admission was similar across all preterm infant categories (*p* = 0.97), smaller centers chose significantly higher temperature levels than centers with higher patient volume (*p* = 0.004). The relative humidity values on admission depended on the infant's GA and BW (*p* < 0.001). Again, a relevant inter-center variability could be seen, especially in infants of 30 WOG/1,500 g (variation coefficient 10%) (*p* ≤ 0.001).

## Discussion

Evidence-based guidelines on the appropriate body core temperature for preterm neonates are largely lacking. It was therefore to be expected that the thermal management practices would show some heterogeneity across the different institutions. Surprisingly, however, the target body temperature data collected in this survey showed a very uniform normal range of 36.6–37.4°C (mean ± 2 SD). Likewise, the definition for hypo- and hyperthermia was very similar for all preterm infants, independent of GA and BW.

The unanimously preferred temperature corresponds to the normal range of body temperatures the WHO defines for term infants (4). Recent studies suggest that this temperature target range could be appropriate for preterm infants, as well: In 10 small preterm infants Knobel et al. (5) observed that a body temperature range of 36.8–37.0°C maximized normal heart rate recordings during the first 12 h of life. Another larger, retrospective analysis by the Canadian Neonatal Network (6) reported a *U*-shaped relationship between body temperature (on admission to the NICU) and adverse outcome with the lowest adverse outcome rates occurring at admission temperatures of 36.5–37.2°C. A more recent study by the NICHD Neonatal research network (17) still found a decrease of in-hospital mortality with increasing admission temperatures even though the latter had risen in the past decade.

However, these studies refer to the conditions of primary care in the delivery room and/or admission to the NICU, respectively, and do not take into account the chronic exposure of preterm babies to potentially suboptimal thermal conditions during their long-lasting hospital stay. The usual textbook recommendations on thermal care on the NICU focus on “thermoneutrality” (14, 15). The thermoneutral temperature is defined as the *ambient* temperature that prevents any thermoregulatory (heat or cold) defense reaction resulting in an increase in metabolic rate (18). In view of the fact that the metabolic rate cannot be routinely measured in preterm neonates, a stable *body* temperature (and, occasionally, a low core-to-shell temperature gradient) is commonly used as a surrogate of thermoneutrality (19). However, the assumption that 37.0°C is the body core temperature that reflects thermoneutrality in preterm neonates irrespective of GA and BW (which might have been reasonable at a time when even this was hard to achieve), has never been endorsed by scientific data.

The consensus on target temperature in preterm infants, as found in our survey, deviates from the intrauterine temperature of the fetus which is assumed to be 0.5–1.0°C higher than that of the mother (which itself is slightly elevated during pregnancy) (1–3, 20). This would correspond to a fetal body temperature of at least 37.5°C. The WHO even describes 38.0°C as the prevalent fetal core temperature (4). In a recent study by Topaloglu et al. (21) the mean rectal body temperature of newborn babies immediately after delivery was found to be 0.4–1.2°C higher than the mother's mean temperature, depending on the mode of delivery. In the vaginal delivery group the babies had a mean rectal temperature of 37.5 ± 0.6°C compared to the mean maternal temperature of 36.3 ± 0.3°C. As has been shown using telemetric methods in pregnant sheep, a core temperature gradient between the fetal and the maternal organism seems to be a characteristic of mammalian pregnancy (22, 23). The heat produced by the fetus has to be removed either through the amniotic fluid and the uterine wall (conductive pathway) or via the umbilical arterial blood flow to the placenta (convective pathway). As the heat conductivity of the placenta is limited and its total resistance to heat flow is larger than zero, fetal temperature exceeds maternal temperature by about 0.5 (0.3–1.0)°C (2, 24).

Keeping these intrauterine conditions in mind, the homogeneity of target temperature values, as found in our survey, is far from being self-evident. Under the designated target temperature range even infants born at 24 WOG who under normal circumstances would remain another 4 months *in utero*, are kept in the incubator at relevantly lower temperatures than physiologically prevalent at this stage of their development. Notably, the lower limit of our identified normal range (36.6°C) is more than 1.0°C lower than the “breeding temperature” that the fetus would be exposed to *in utero* (37.5–38.0°C).

Up to now, there are no animal and only a few clinical studies having tested higher target values for body temperature in preterm neonates. In a small observational study with 14 VLBWI, it was shown that when treated under conventional thermoneutral conditions, the infants' vasomotor tone, a surrogate for cold stress, was high. When the infants' body temperatures were raised to 37.5–38.5°C the vasomotor tone was reduced and less problems were reported by the designated nursing staff (25). In a more recent study on 38 incubated preterm infants during their first 11 days of life, even a body temperature of 37.0°C was associated with considerably lower energy costs and greater weight gain, as compared with the 36.8°C attained by a different incubator mode (26).

The lack of scientific evidence contrasts to the considerable efforts to assess the optimal target values for other physiological parameters such as oxygen saturation (27) or carbon dioxide tension (28) that have been made in the past few years. Thus, thermal care appears to be a somewhat neglected neonatological practice that merits further study, not only with regard to short-term outcomes such as weight gain and metabolic stress, but also to potential long-term consequences such as neurological development.

On the other hand, our results show that awareness for a higher thermal instability of the preterm infant does exist. The primary regulation of incubators was conducted choosing higher air temperature and relative humidity levels for more premature infants, a practice in line with conventional textbook recommendations (13–15). Moreover, the cut-off point for hypothermia was defined at a slightly higher body temperature value, the more immature the infant is, although this difference did not reach statistical significance. Consistent with the findings of a French study from 2012 (16) a small inter-center variability in the choice of air temperatures was present, in the choice of humidity values it was high. This variation underlines the lack of binding evidence-based guidelines in the thermal management of small preterm infants. Although most centers refer to standard operating procedures (SOPs), 42% of them base their decisions regarding temperature targets and thermal management on the clinical assessment of the caretaker at bedside only.

Another remarkable finding of the study is that the majority (68%) of participating NICUs prefer the ATC over the SSC mode of incubator operation, revealing the typical national practices in neonatal care. Moreover, only 16% of all centers report the use of central-peripheral gradient monitoring which has repeatedly been proposed not only as an appropriate indicator of thermal comfort (13, 25), but also as an early predictor of late-onset sepsis in VLBWI (29, 30).

Our survey seemed to be well-structured and comprehensive. This is reflected in the satisfactory quota of complete responses even though the overall response rate (notably from Austrian NICUs) remained below our expectations. However, with a total of 87 eligible responses (out of 149 addressed perinatal centers, i.e., 58%) from Germany and Switzerland, it can be considered as representative for high-level perinatal centers in these countries.

The study has several other limitations: In VLBWI evaporative heat loss is the predominant mode of heat loss during the first few days of life, which then decreases with increasing postnatal age. The high ambient temperatures and relative humidities initially needed to maintain an adequate body temperature in VLBWI can thus be gradually reduced over time. As we asked for incubator settings on admission only, we did not capture the course of applied ambient temperatures and relative humidities with increasing postnatal age of the infants.

Inherent to the survey instrument, a potential social-desirability bias cannot be ruled out especially since the one responding was often not identical with the staff members responsible for thermal care at bedside. Moreover, as temperature ranges and cut-off points for hypo- and hyperthermia were provided in preset temperature intervals and values, the participants could only select temperature or humidity values differing from another in 0.5°C or 5% intervals, respectively. Also the given premature subgroups may have confused participants, not knowing exactly where to place a premature baby lying somewhere in between. The use of free text boxes might have led to a larger variation of results.

## Conclusion

In conclusion, as revealed by this survey, there is a tacit consensus among high-level NICUs that 37.0°C is the normal and thus desirable body temperature for VLBWI, irrespective of gestational age and/or birth weight. This is in contrast to the fact that the intrauterine fetal body temperature is assumed to be 0.5-1.0°C higher than the maternal one and has not been assessed by prospective clinical studies relating target body temperature to short- and long-term outcomes.

## Data Availability

The datasets generated for this study are available on request to the corresponding author.

## Author Contributions

AP and FvdM contributed equally to the development of the questionnaire, analysis of the data, and preparation of the manuscript. DS was involved in the planning of the study, discussion of the results, and finalization of the paper.

### Conflict of Interest Statement

The authors declare that the research was conducted in the absence of any commercial or financial relationships that could be construed as a potential conflict of interest.
