# Supplementary material for: Target Body Temperature in Very Low Birth Weight Infants: Clinical Consensus in Place of Scientific Evidence
Source: Front Pediatr. 2019 Jun 7;7:227. doi: 10.3389/fped.2019.00227 (PMC6568209; doi:10.3389/fped.2019.00227)
Supplement: Supplementary file 1 [file Table_1.DOCX]

|  | GA [WOG] / BW [g] | Mean [°C] | SD [°C] | Range (± 2 SD) |
| --- | --- | --- | --- | --- |
| Target Body Temperature | 30 / 1500 | 36.98 | 0.17 | 36.64 – 37.32 |
|  | 27 / 1000 | 36.99 | 0.22 | 36.55 – 37.43 |
|  | 24 / 500 | 37.02 | 0.20 | 36.62 – 37.42 |
| Limit of  Hypothermia | 30 / 1500 | 36.44 | 0.27 | 35.90 – 36.98 |
|  | 27 / 1000 | 36.51 | 0.28 | 35.95 – 37.07 |
|  | 24 / 500 | 36.54 | 0.30 | 35.94 – 37.14 |
| Limit of Hyperthermia | 30 / 1500 | 37.53 | 0.28 | 36.97 – 38.09 |
|  | 27 / 1000 | 37.53 | 0.30 | 36.93 – 38.13 |
|  | 24 / 500 | 37.52 | 0.31 | 36.90 – 38.14 |

**Supplementary Table 1** Body temperature targets and limits of hypo-/hyperthermia in three different categories of VLBW (original data to Figs. 1 and 2).
