# Supplementary material for: Target Body Temperature in Very Low Birth Weight Infants: Clinical Consensus in Place of Scientific Evidence
Source: Front Pediatr. 2019 Jun 7;7:227. doi: 10.3389/fped.2019.00227 (PMC6568209; doi:10.3389/fped.2019.00227)

Sehr geehrte(r) Umfrage-Teilnehmer(in),

wir möchten Sie darauf aufmerksam machen, dass es für Sie zu jedem Zeitpunkt während dieser Umfrage die Möglichkeit gibt, über den Funktionsbutton "Zurück" am Unterrand jeder Seite, Ihre Antworten nochmals zu überprüfen und ggf. zu korrigieren.

Viel Vergnügen bei der Beantwortung unserer Fragen und herzlichen Dank für Ihre Teilnahme!

Allgemeines

1. Bitte geben Sie die Postleitzahl Ihrer Klinik ein:

2. Wie viele Betten hat Ihre Früh- und Neugeborenen-Intensivstation?

3. Welcher Berufsgruppe gehören Sie an?

- ○ Pflege(fach)kraft
- ○ Pflegerische Leitung (Stationsleitung)
- ○ Stationsarzt
- ○ Ärztliche Leitung (Oberarzt, Chefarzt)

4. Welche Berufsgruppe ist in Ihrer Abteilung im klinischen Alltag HAUPTSÄCHLICH verantwortlich für das Thermomanagement bei Frühgeborenen?

- ○ Pflege(fach)kraft am Patientenbett
- ○ Stationsleitung Pflege
- ○ Stationsarzt
- ○ Ärztliche Leitung (Oberarzt, Chefarzt)

5. Wie viele Frühgeborene mit einem Geburtsgewicht < 1500g (Very Low Birth Weight, VLBW) werden pro Jahr auf Ihrer neonatologischen Intensivstation im Durchschnitt behandelt ?

Temperatur - Ziel

6. Was ist der Zielbereich für die Körperkerntemperatur von Frühgeborenen auf Ihrer Abteilung?

Erklärung: Hier ist es möglich, mehrere Haken pro Zeile zu setzen.
Beispiel: Ihr Zielbereich für die Körperkerntemperatur für Frühgeborene der 30. SSW liegt bei 36,5-37,5 °C. Dann setzen Sie bitte in die Zeile 30 SSW, 1500g einen Haken bei 36,5°C, 37°C und 37,5°C.
Entsprechend verfahren Sie für die anderen beiden Frühgeborenen-Kategorien.

	36,0°C	36,5°C	37,0°C	37,5°C	38,0°C
30 SSW, 1500g	☐	☐	☐	☐	☐
27 SSW, 1000g	☐	☐	☐	☐	☐
24 SSW, 500g	☐	☐	☐	☐	☐

7. Bei wie viel °C gemessener Körperkerntemperatur definieren Sie eine Hypothermie und veranlassen eine Maßnahme zur Gegenregulation?
Hier ist jeweils eine Antwort pro Zeile zulässig.

	< 36,0°C	< 36,5°C	< 37,0°C
30 SSW, 1500g	☐	☐	☐
27 SSW, 1000g	☐	☐	☐
24 SSW, 500g	☐	☐	☐

8. Bei wie viel °C gemessener Körperkerntemperatur definieren Sie eine Hyperthermie und veranlassen eine Maßnahme zur Gegenregulation? Hier ist jeweils eine Antwort pro Zeile zulässig.

	> 37,0°C	> 37,5°C	> 38,0°C
30 SSW, 1500g	☐	☐	☐
27 SSW, 1000g	☐	☐	☐
24 SSW, 500g	☐	☐	☐

Temperatur - Messung

9. Wie erfolgt auf Ihrer Abteilung die Körpertemperaturmessung der Frühgeborenen? HAUPTSÄCHLICH...

- ◯ kontinuierlich
- ◯ intermittierend

Kontinuierliche Temperaturmessung

10. Bitte geben Sie die HAUPTSÄCHLICH verwendete Messmethode an

- ◯ Rektalsonde
- ◯ Hautsonde zentral Abdomen
- ◯ Hautsonde zentral Rücken/Liegefläche
- ◯ Hautsonde zentral und peripher = Gradientenmessung (z.B. Abdomen und Fußsohle)
- ◯ Sonstiges (bitte angeben)

IntermittierendeTemperaturmessung

11. Bitte geben Sie die HAUPTSÄCHLICH verwendete Messmethode an.

- ◯ Rektal per Digitalthermometer
- ◯ Axillär per Digitalthermometer
- ◯ Sonstiges (bitte angeben)

Gradientenmessung (z.B. Abdomen und Fußsohle)

12. Was ist der Zielbereich für gemessene Temperatur-Gradienten von Frühgeborenen auf Ihrer Abteilung?

Erklärung: Hier ist es möglich, mehrere Haken pro Zeile zu setzen.
Beispiel: Sie definieren Ihren Zielbereich für den Temperatur-Gradienten bei Frühgeborenen der 30. SSW bis maximal 1,5°C. Dann setzen Sie bitte in die Zeile 30 SSW, 1500g einen Haken bei
≤ 0,5°C , ≤ 1°C und ≤ 1,5°C.
Entsprechend verfahren Sie für die anderen beiden Frühgeborenen-Kategorien.

	≤ 0,5 °C	≤ 1,0 °C	≤ 1,5 °C	≤ 2,0 °C
30 SSW, 1500g	☐	☐	☐	☐
28 SSW, 1000g	☐	☐	☐	☐
24 SSW, 500g	☐	☐	☐	☐

Geräte zur Wärmetherapie

13. Welches Gerät wird auf Ihrer Abteilung zur Temperaturregulierung von Frühgeborenen HAUPTSÄCHLICH verwendet?

- ◯ Wärmestrahler
- ◯ Inkubator

Wärmetherapie mittels Inkubator - Modus

14. Welcher Temperatur-Modus am Inkubator wird auf Ihrer Abteilung HAUPTSÄCHLICH verwendet?

- ◯ Lufttemperaturregulierung (manueller Modus)
- ◯ Hauttemperaturregulierung (Servo Control Modus)

Wärmetherapie mittels Inkubator - Lufttemperaturregulierung (manueller...

15. Was ist der Zielbereich für die eingestellte Lufttemperatur im Inkubator von Frühgeborenen BEI AUFNAHME auf Ihre Abteilung?

Erklärung: Hier ist es möglich, mehrere Haken pro Zeile zu setzen.
Beispiel: Ihr Zielbereich für die Lufttemperatur im Inkubator liegt bei Frühgeborenen der 30. SSW zwischen 35,0°C-37,0°C. Dann setzten Sie bitte Ihre Haken in der Zeile 30 SSW, 1500g bei 35,0°C, 36,0°C und 37,0°C. Entsprechend verfahren Sie für die anderen beiden Frühgeborenen-Kategorien.

	32,0°C	33,0°C	34,0°C	35,0°C	36,0°C	37,0°C	38,0°C
30 SSW, 1500g	☐	☐	☐	☐	☐	☐	☐
27 SSW, 1000g	☐	☐	☐	☐	☐	☐	☐
24 SSW, 500g	☐	☐	☐	☐	☐	☐	☐

16. Was ist der Zielbereich für die eingestellte Luftfeuchtigkeit im Inkubator von Frühgeborenen BEI AUFNAHME auf Ihre Abteilung?

Erklärung: Hier ist es möglich, mehrere Haken pro Zeile setzen.
Beispiel: Ihr Zielbereich für die Luftfeuchtigkeit im Inkubator liegt bei Frühgeborenen der 30. SSW zwischen 55-70%. Dann setzten Sie bitte Ihre Haken in der Zeile 30 SSW, 1500g bei 55%, 60%, 65% und 70%. Entsprechend verfahren Sie für die anderen beiden Frühgeborenen-Kategorien.

	50%	55%	60%	65%	70%	75%	80%
30 SSW, 1500g	☐	☐	☐	☐	☐	☐	☐
27 SSW, 1000g	☐	☐	☐	☐	☐	☐	☐
24 SSW, 500g	☐	☐	☐	☐	☐	☐	☐

17. Was ist der Zielbereich für die Hauttemperatur von Frühgeborenen BEI AUFNAHME auf Ihre Abteilung?

Erklärung: Hier ist es möglich, mehrere Haken pro Zeile setzen.
Beispiel: Ihr Zielbereich für die Hauttemperatur bei Frühgeborenen der 30. SSW liegt zwischen 35,5°C-37,0°C. Dann setzten Sie bitte Ihre Haken in der Zeile 30 SSW, 1500g bei 35,5°C, 36,0°C, 36,5°C und 37,0°C.
Entsprechend verfahren Sie für die anderen beiden Frühgeborenen-Kategorien.

	35,0	35,5	36,0	36,5	37,0	37,5	38,0
30 SSW, 1500g	☐	☐	☐	☐	☐	☐	☐
27 SSW, 1000g	☐	☐	☐	☐	☐	☐	☐
24 SSW, 500g	☐	☐	☐	☐	☐	☐	☐

18. Was ist der Zielbereich für die eingestellte Luftfeuchtigkeit im Inkubator von Frühgeborenen BEI AUFNAHME auf Ihre Abteilung?

Erklärung: Hier ist es möglich, mehrere Haken pro Zeile setzen.
Beispiel: Ihr Zielbereich für die Luftfeuchtigkeit im Inkubator liegt bei Frühgeborenen der 30. SSW zwischen 55-70%. Dann setzten Sie bitte Ihre Haken in der Zeile 30 SSW, 1500g bei 55%, 60%, 65% und 70%.
Entsprechend verfahren Sie für die anderen beiden Frühgeborenen-Kategorien.

	50%	55%	60%	65%	70%	75%	80%
30 SSW, 1500g	☐	☐	☐	☐	☐	☐	☐
27 SSW, 1000g	☐	☐	☐	☐	☐	☐	☐
24 SSW, 500g	☐	☐	☐	☐	☐	☐	☐

Wärmetherapie mittels Wärmestrahler

19. Was ist der Zielbereich für die zentral gemessene Temperatur (z.B. Hauttemperatur, Rektaltemperatur) von Frühgeborenen BEI AUFNAHME auf Ihre Abteilung?

Erklärung: Hier ist es möglich, mehrere Haken pro Zeile setzen.
Beispiel: Ihr Zielbereich für die zentral gemessene Temperatur bei Frühgeborenen der 30. SSW liegt bei 36.5°C-37,5°C. Dann setzen Sie bitte in der Zeile 30 SSW, 1500g einen Haken bei 36,5°C, 37,0°C und 37,5°C.
Entsprechend verfahren Sie für die anderen beiden Frühgeborenen-Kategorien.

	35,0°C	35,5°C	36,0°C	36,5°C	37,0°C	37,5°C	38,0
30 SSW, 1500g	☐	☐	☐	☐	☐	☐	☐
27 SSW, 1000g	☐	☐	☐	☐	☐	☐	☐
24 SSW, 500g	☐	☐	☐	☐	☐	☐	☐

Entscheidungsgrundlagen

20. Auf welcher Grundlage werden Entscheidungen bezüglich des Thermoregulationsmodus hauptsächlich getroffen?

- ◯ SOP / abteilungseigener Standard
- ◯ Empfehlung des Herstellers
- ◯ Empfehlung aus der wissenschaftlichen Literatur
- ◯ Klinische Einschätzung durch die betreuende Pflegefachkraft am Bett

Zusätzliche wärmetherapeutische Maßnahmen

21. Welche zusätzlichen wärmetherapeutischen Maßnahmen (in Kombination mit den hauptsächlich eingesetzten Geräten) benutzen Sie (Mehrfachantworten möglich)?

- ☐ Plastikfolie auf der Erstversorgungseinheit im Kreißsaal
- ☐ Ergänzender Wärmestrahler neben der Erstversorgungseinheit im Kreißsaal
- ☐ Plastikfolie auf der offenen Einheit auf der Intensivstation
- ☐ Plastikfolie im Inkubator auf der Intensivstation
- ☐ Ergänzender Wärmestrahler bei Manipulationen im Inkubator auf der Intensivstation
- ☐ Wärmestrahler auf offener Einheit

Sonstiges (bitte angeben)

[ ]

Sie haben vor dem Abschicken der Umfrage die Möglichkeit, über den Funktionsbutton "Zurück" am Unterrand jeder Seite, alle Ihre Antworten nochmals zu überprüfen und ggf. zu korrigieren.

Herzlichen Dank für Ihre Beteiligung an unserer Umfrage!

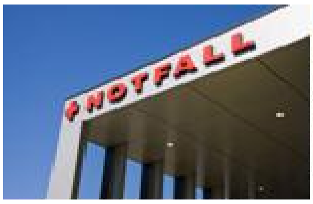

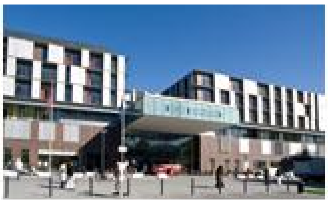
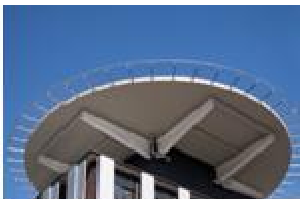

Supplement: Supplementary file 2 [file Data_Sheet_1.PDF]
