# Supplementary material for: Target Body Temperature in Very Low Birth Weight Infants: Clinical Consensus in Place of Scientific Evidence
Source: Front Pediatr. 2019 Jun 7;7:227. doi: 10.3389/fped.2019.00227 (PMC6568209; doi:10.3389/fped.2019.00227)
Supplement: Supplementary file 3 [file Data_Sheet_2.PDF]

Survey English Version

Dear participant,

please note that you can check and, if necessary, correct your answers at any point in this questionnaire by clicking the “back” button on the lower border of each page.

We hope you enjoy answering the questions, and thank you very much for your participation!

General

1. Please provide your clinic’s zip/postal code.

.....

2. How many beds does your newborn intensive care unit (NICU) have?

.....

3. What staff position do you hold?

- (Certified) nurse
- Head nurse (ward head)
- Ward physician
- Head physician (senior physician, department head)

4. Which staff member in your department is MAINLY responsible for the thermal management of premature infants in daily clinical routine?

- Bedside nurse
- Head nurse (ward head)
- Ward physician
- Head physician (senior physician, department head)

5. How many premature infants weighing less than 1500g at birth (Very Low Birth Weight, VLBW) are treated on average per year at your NICU?

.....

Temperature Target

6. What range of body core temperature for premature infants are you aiming at in your department?

You can tick more than one box in the following table. If, for example, the body core temperature of premature infants born at 30 weeks of gestation should be in the range of 36.5°C-37.5°C, then tick the boxes under each of 36.5°C, 37°C and 37.5°C in the line “30 GA/PMA, 1500g”. Proceed accordingly for the other two premature infant categories.

	36,0°C	36,5°C	37,0°C	37,5°C	38,0°C
30 WOG, 1500g					
27 WOG, 1000g					
24 WOG, 500g					

7. At what body core temperature (how many degrees Celsius) do you define hypothermia and initiate treatment measures to counterregulate it?

Here, only one answer per line is permitted.

	< 36,0°C	< 36,5°C	< 37,0°C
30 WOG, 1500g			
27 WOG, 1000g			
24 WOG, 500g			

8. At what body core temperature (how many degrees Celsius) do you define hypothermia and initiate treatment measures to counterregulate it?

Here, only one answer per line is permitted.

	> 37,0°C	> 37,5°C	> 38,0°C
30 WOG, 1500g			
27 WOG, 1000g			
24 WOG, 500g			

Temperature Measurement

9. How is the body temperature of premature infants measured in your department? MAINLY…

- continuously
- intermittently

Continuous Temperature Measurement

10. Please indicate the PRINCIPALLY employed measurement method.

- Rectal sensor
- Skin sensor placed on central abdomen
- Skin sensor placed on back/area being laid on
- Central and peripheral skin sensors (e.g. abdomen and sole of foot), i.e. gradient measurement
- Other (please indicate)

Intermittent Temperature Measurement

11. Please indicate the PRINCIPALLY employed measurement method.

- Rectally with digital thermometer
- Axillary with digital thermometer
- Other (please indicate)

Gradient Measurement (e.g. abdomen and sole of foot)

12. What range of temperature gradients for premature infants are you aiming at in your department?
You can tick more than one box in the following table. If, for example, the temperature gradient of premature infants born at 30 weeks of gestation should NOT exceed a maximum of 1.5°C, then tick the boxes under each of ≤ 0.5°C, ≤ 1°C and ≤ 1.5°C in the line "30 GA/PMA, 1500g". Proceed accordingly for the other two premature infant categories.

	≤ 0,5°C	≤ 1,0°C	≤ 1,5°C	≤ 2,0°C
30 WOG, 1500g				
27 WOG, 1000g				
24 WOG, 500g				

Heat therapy devices

13. Which device is MAINLY used for temperature regulation of premature infants in your department ?

- Heat radiator
- Incubator

Heat Therapy via Incubator

14. Which temperature mode is MAINLY used for incubators in your department?

- Air temperature regulation (manual mode)
- Skin temperature regulation (Servo Control mode)

Heat Therapy via Incubator - Air Temperature Regulation (Manual Mode)

15. AT ADMISSION, what range of incubator air temperatures are you aiming at for premature infants in your department?

You can tick more than one box in the following table. If, for example, the incubator air temperature for premature infants born at 30 weeks of gestation should be between 35.0°C and 37.0°C, then tick the boxes under each of 35.0°C, 36.0°C and 37.0°C in the line "30 GA/PMA, 1500g". Proceed accordingly for the other two premature infant categories.

	32,0°C	33,0°C	34,0°C	35,0°C	36,0°C	37,0°C	38,0°C
30 WOG, 1500g							
27 WOG, 1000g							
24 WOG, 500g							

16. AT ADMISSION, what range of air humidity values do you set in the incubator for a premature infant in your department?

You can tick more than one box in the following table. If, for example, the incubator air humidity for premature infants born at 30 weeks of gestation should range between 55% and 70%, then tick the boxes under each of 55%, 60%, 65% and 70% in the line "30 GA/PMA, 1500g". Proceed accordingly for the other two premature infant categories.

	50%	55%	60%	65%	70%	75%	80%
30 WOG, 1500g							
27 WOG, 1000g							
24 WOG, 500g							

Heat Therapy via Incubator - Skin Temperature Regulation (Servo Control Mode)

17. AT ADMISSION, what range of skin temperatures are you aiming at for premature infants in your department?

    You can tick more than one box in the following table. If, for example, you want premature infants born at 30 weeks of gestation to have a skin temperature between 35.5°C and 37.0°C, then tick the boxes under each of 35.5°C, 36.0°C, 36.5°C and 37.0°C in the line "30 GA/PMA, 1500g". Proceed accordingly for the other two premature infant categories.

	35,0°C	35,5°C	36,0°C	36,5°C	37,0°C	37,5°C	38,0°C
30 WOG, 1500g							
27 WOG, 1000g							
24 WOG, 500g							

18. AT ADMISSION, what range of air humidity values do you set in the incubator for a premature infant in your department?

    You can tick more than one box in the following table. If, for example, the air humidity in an incubator for premature infants born in the 30th PW should be between 55% and 70%, then tick the boxes under each of 55%, 60%, 65% and 70% in the line "30 GA/PMA, 1500g". Proceed accordingly for the other two premature infant categories.

	50%	55%	60%	65%	70%	75%	80%
30 WOG, 1500g							
27 WOG, 1000g							
24 WOG, 500g							

Heat Therapy via Radiator

19. AT ADMISSION, what range of centrally measured temperature (e.g. skin or rectal temperature) for premature infants are you aiming at in your department?

You can tick more than one box in the following table. If, for example, the centrally measured temperature for premature infants born at 30 weeks of gestation should be in the range of 36.5°C to 37.5°C, then tick the boxes under each of 36.5°C, 37.0°C and 37.5°C in the line “30 GA/PW, 1500g”. Proceed accordingly for the other two premature infant categories.

	35,0°C	35,5°C	36,0°C	36,5°C	37,0°C	37,5°C	38,0°C
30 WOG, 1500g							
27 WOG, 1000g							
24 WOG, 500g							

Decision Basis

20. On what basis, are decisions concerning thermal regulation of the premature infant MAINLY made in your department?

- Standard Operating Procedure (SOP)/ department-specific standard
- Recommendation of the manufacturer
- Recommendation from the scientific literature
- Clinical assessment of the bedside nursing staff

Additional heat therapy measures/devices

21. Which additional heat therapy devices do you employ (in combination with the mainly used devices)?

- Polyethylene wrap/bag on the primary care unit in the delivery room
- Additional heat radiator on the primary care unit in the delivery room
- Polyethylene wrap/bag on the open cot in the NICU
- Polyethylene wrap/bag in the incubator in the NICU
- Additional heat radiator during manipulations in the incubator in the NICU
- Additional heat radiator on open cot in the NICU
- Other (please indicate)

Before sending off the questionnaire you can use the function button „back“ on the lower border of each page to check on your answers and, if necessary, correct them.

Thank you very much for your participation in our survey!
